# Case Report: Neuroendocrine carcinoma of the breast: a review of the literature and illustration of six cases

**DOI:** 10.3389/fmed.2025.1551309

**Published:** 2025-08-20

**Authors:** Nazire E. Albayrak

**Affiliations:** Department of Pathology, Molecular and Cell-Based Medicine, Icahn School of Medicine at Mount Sinai, New York, NY, United States

**Keywords:** neuroendocrine carcinoma, breast neoplasms, neuroendocrine tumors, mTOR inhibitors, synaptophysin

## Abstract

“Primary neuroendocrine breast carcinoma (NEBC) is an underdiagnosed subtype of breast cancer, which includes small cell (SCNEC) and large cell neuroendocrine carcinomas (LCNEC). Accurate diagnosis remains challenging given their low incidence; misclassification as invasive breast carcinoma of no special type (IBC-NST), invasive ductal carcinoma (IDC), or a metastatic neuroendocrine carcinoma may occur. Cases with any component of adenocarcinoma and well-differentiated neuroendocrine tumors were excluded. A search of the pathology database (2012–2024) revealed six female patients (27–85 years) with a final pathologic diagnosis of NEBC (stages IA–IV), including four diagnosed with LCNEC and two with SCNEC. Even though most NEBC cases (5 of 6; 83%) were of the luminal subtype, five of six patients (83%) developed distant metastases within 4 years of the initial diagnosis. Molecular profiling of six cases revealed common alterations in the FGF/FGFR and PI3K/AKT/mTOR pathways. In summary, primary neuroendocrine carcinomas of the breast display aggressive behavior. However, they are more likely to harbor certain alterations, such as activating *PIK3CA* mutations and *FGFR1* amplification, which can be of therapeutic value. The Ki-67 index, unlike in the pancreas and gastrointestinal tract, is not suitable for grading neuroendocrine neoplasms (NENs) of the breast. However, it can still serve as a tool for risk stratification, similar to its use in luminal-type breast cancer.

## 1 Introduction

Primary neuroendocrine breast carcinomas (NEBCs) represent a rare diagnostic entity, with an incidence ranging from 0.1 to 5% (1, 2). Formal diagnostic criteria for NEBCs were first established in 2003 by the WHO as a classification of tumors having >50% neoplastic cells expressing neuroendocrine markers (3). In the 2012 revision, the threshold value of > 50% neuroendocrine marker expression was removed (1). Breast tumors with neuroendocrine differentiation were classified into three groups as follows: well-differentiated neuroendocrine tumor, poorly differentiated/small cell neuroendocrine carcinoma (SCNEC), and invasive breast carcinoma with neuroendocrine differentiation. The third group included invasive breast carcinoma of no special type (IBC-NST) as well as special types, such as solid papillary carcinoma and the hypercellular variant of mucinous carcinoma. Further studies, however, have shown that neuroendocrine differentiation by immunohistochemistry (IHC) is observed in up to 20% of mammary carcinomas (4, 5). Therefore, a key feature of the current revision, the fifth edition of the WHO Classification of Breast Tumors, is the exclusion of specific histologic types, including IBC-NSTs with neuroendocrine differentiation, and the inclusion of large cell neuroendocrine carcinomas (LCNECs), which more accurately reflects the prevalence of NEBCs (6).

## 2 Materials and methods

Ethical approval and patient consent were not sought for this retrospective study, as the cases used in this case series have been completely de-identified, and no additional tests were performed beyond the diagnostic workup, for which informed consent was obtained from patients for each diagnostic procedure. A search of the pathology database from 2012 to 2024 revealed 17 patients with a final diagnoses of primary neuroendocrine neoplasms (NENs) of the breast, representing 0.18% of all registered breast cancer cases (*n* = 9,068) during 2012–2024. According to the current WHO criteria (6), the tumors for 7 patients were classified as primary neuroendocrine tumors (NETs) of the breast with low to intermediate-grade morphology, while 10 patients were diagnosed with poorly differentiated NEBCs, corresponding to a prevalence of 0.1%. Well to moderately-differentiated neuroendocrine tumors and invasive breast carcinoma with less than 90% of cells expressing neuroendocrine markers were excluded. Four cases of NEBCs had to be excluded, as histologic specimens could not be retrieved for reevaluation. The remaining six cases had complete clinical follow-up at our institution.

Foundation One test was performed on formalin-fixed paraffin-embedded or whole-blood-driven samples from six patients diagnosed with NEBCs as part of clinical practice. Foundation One, a target-specific next-generation sequencing (NGS)-based device from Foundation Medicine, is capable of detecting 324 molecular alterations, including substitutions, indels, copy number alterations, selected genomic rearrangements, and genomic signatures, such as tumor fraction, blood tumor mutational burden, and microsatellite instability status (7).

A comprehensive literature review was conducted via PubMed search using a combination of keywords: ‘neuroendocrine carcinoma of the breast’, ‘primary neuroendocrine breast carcinoma’, ‘small cell neuroendocrine carcinoma of the breast’, ‘histology’, ‘immunohistochemical profiling’, ‘WHO Classification of neuroendocrine neoplasms of the breast’, ‘management’, ‘prognosis’, and ‘molecular characteristics’. The selected studies were reviewed for clinicopathological characteristics, including TNM staging, therapeutic strategies, prognostic information, immunohistochemical features, and genomic landscape. Well-differentiated neuroendocrine tumors, as well as special types of breast tumors with neuroendocrine differentiation and indolent behavior (i.e., solid papillary carcinoma and hypercellular-subtype mucinous carcinoma), were excluded from the review process.

## 3 Results

Clinicopathologic characteristics, including molecular characterization of the six cases, are provided in Table 1.

**Table 1:** Clinicopathological features of six patients with NEBC.

Patient no.	Diagnosis	Primary tumor size	Axillary node status	Distant metastases	Ki-67	Intrinsic subtype	Immunohistochemistry	Molecular work-up
1	Large cell neuroendocrine carcinoma	30 mm	N/A	Pulmonary	25%	90%ER, 40%PR, HER2- (Luminal A)	Synaptophysin+, Chromogranin-, INSM1-, CD56-, GATA3+, TTF-1-, CDX-2-, PAX8-	5 alterations: *TP53, PIK3CA, GATA3, CCND2, MYC*
2	Small cell neuroendocrine carcinoma	20 mm	11/12	Contralateral breast	40%	0%ER, 0%PR, HER2- (Basal-like)	Synaptophysin+, Chromogranin-, INSM1+, CD56+, CK5/6+, CK7-, CK20-, GATA3-, Mammaglobin-, TTF-1-, CDX-2-, PAX8-	6 alterations: *TP53, FGFR1, AKT1, KMT2D, KRAS, RB1*
3	Small cell neuroendocrine carcinoma	63 mm	Positive: left axillary lymphadenopathy	Pulmonary, Osseous	50%	95%ER, 75%PR, HER2 + (Luminal B, HER2-positive)	Synaptophysin+, Chromogranin-, INSM1+, CD56+, p40-, GATA3+, TTF-1-, CDX-2-, PAX8-	6 alterations: *TP53, FGFR2, ERBB2, CCNE1, MYC, PPARG*
4	Large cell neuroendocrine carcinoma	17 mm	Negative	None	80%	95%ER, 5%PR, HER2- (Luminal B, HER2-negative)	Synaptophysin+, Chromogranin-, INSM1-, CD56-, SMA-, desmin-, p63-, S100-, Mammaglobin+, E-cadherin+, GATA3+, TTF-1-, CDX-2-, PAX8-	Wild-type
5	Large cell neuroendocrine carcinoma	68 mm	N/A	Brain, Osseous, Hepatic	20%	90%ER, 20%PR, HER2- (Luminal A)	Synaptophysin+, Chromogranin+, INSM1+, CD56+, CK7+, CK20-, GATA3+, TTF-1-, CDX-2-, PAX8-	4 alterations: *TP53, FGFR1, PIK3CA, MAP2K4*
6	Large cell neuroendocrine carcinoma	95 mm	N/A	Brain, Pulmonary, Pleural, Pericardial, Osseous, Hepatic	40%	85%ER, 5%PR, HER2- (Luminal B, HER2-negative)	Synaptophysin+, Chromogranin+, INSM1+, CD56+, GATA3+, BRST2+, mammaglobin-, TTF-1-, CDX-2-, PAX8-	3 alterations: *TP53*, *FGF3, CCND1*

NEBC, neuroendocrine breast carcinomas; N/A, not available; ER, estrogen receptor; PR, progesterone receptor; HER2, human epidermal growth factor receptor 2.

### 3.1 Case 1

An 85-year-old woman with a 4-year history of hormone receptor (HR)-positive IBC-NST presented with shortness of breath and altered mental status. A computed tomography (CT) scan of the chest revealed multifocal pneumonia distal to increasing bilateral pulmonary nodules, suggestive of progressive disease. The patient was admitted to the intensive care unit with acute hypoxemic respiratory failure and severe sepsis. Shortly after admission, she died from complications of her post-obstructive pneumonia.

Postmortem breast examination revealed a 3 cm, calcified, white-tan mass in the lower outer quadrant of her right breast. Additional relevant findings included numerous pulmonary nodules.

**Figure 1:**
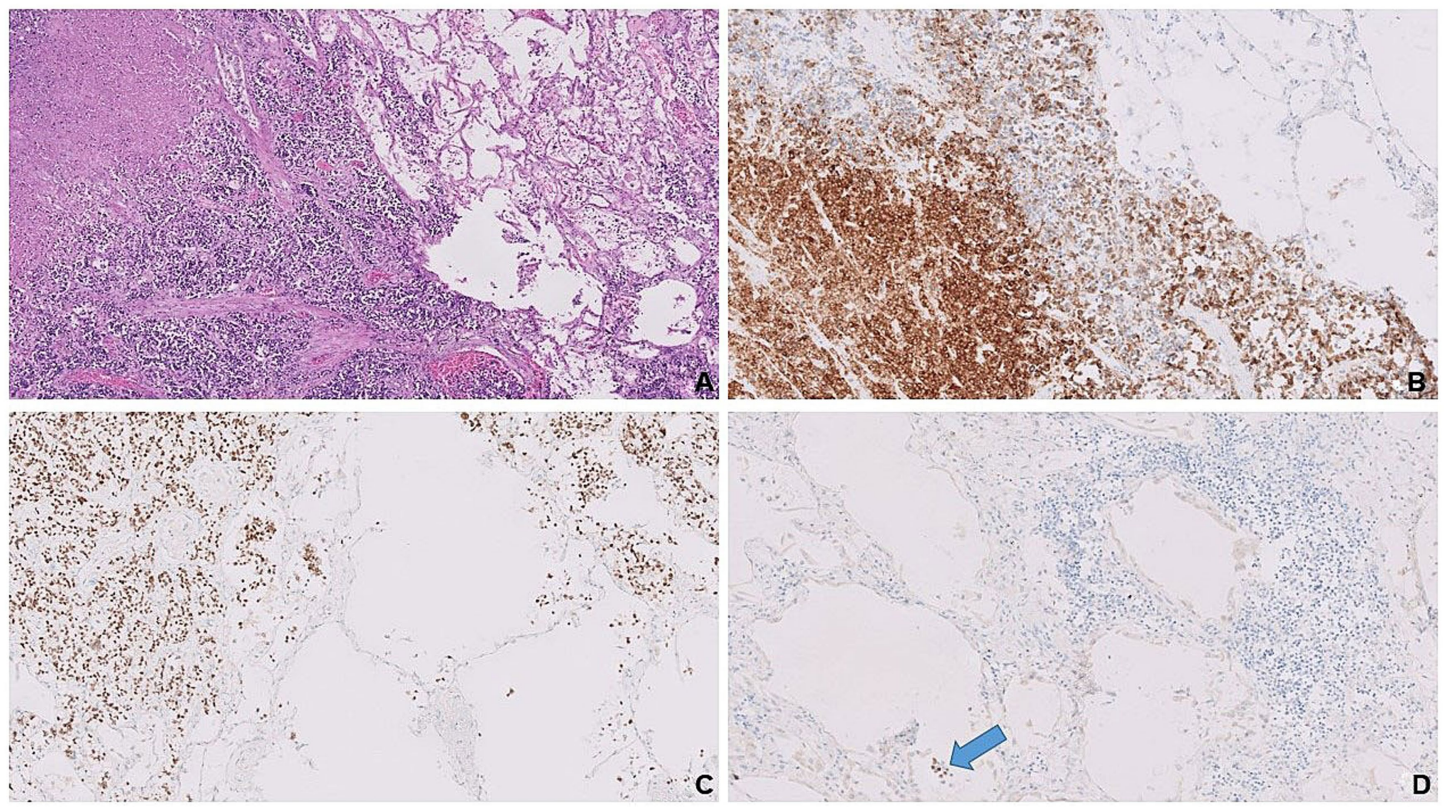
Solid tumor nests adjacent to the benign lung parenchyma on the right. Note geographic necrosis in the left upper corner **(A)**. Tumor cells expressing synaptophysin **(B)** and nuclear GATA3 **(C)** vs. lung parenchyma in between tumor clusters shows no staining. Scattered alveolar pneumocytes (blue arrow) expressing TTF-1 **(D)** as positive internal control vs. no staining in tumor clusters on the right upper corner.

Histological analysis of both the right breast mass and pulmonary nodules showed solid nests and trabeculae of loosely cohesive tumor cells separated by fibrous septa. Associated geographic tumor necrosis was present (Figure 1A). The tumor cells were polygonal with abundant eosinophilic cytoplasm and stippled nuclei with conspicuous nucleoli, reminiscent of LCNEC.

Immunohistochemical analysis demonstrated that the majority of tumor cells expressed synaptophysin (Figure 1B), confirming neuroendocrine differentiation. Since LCNECs commonly arise in the bronchopulmonary or gastroenteropancreatic tracts, immunostaining for TTF-1, CDX2, and PAX8 was performed to rule out metastases from these regions. The tumor cells showed strong nuclear expression for GATA3 (Figure 1C) but no staining for TTF-1 (Figure 1D), CDX2, and PAX8, inferring primary breast origin. Additionally, tumor cells exhibited a luminal A-like phenotype, with positive estrogen receptor (ER) and progesterone receptor (PR) expression and negative human epidermal growth factor receptor 2 (HER2) status.

Molecular testing by targeted NGS revealed clinically significant amplifications of *MYC* and *CCND2* genes, as well as three alterations involving frequently implicated genes in HR-positive breast cancer: *PIK3CA, TP53,* and *GATA3* (8).

### 3.2 Case 2

A 31-year-old woman with a 4-year history of triple-negative right breast cancer presented with a firm, palpable, left breast mass at the 9 o’clock position. She had previously completed neoadjuvant chemotherapy combined with immunotherapy, followed by a modified radical mastectomy.

A month after the surgery, adjuvant radiotherapy was initiated. After 15 cycles of radiotherapy, the patient was found to have a new anterior chest wall lesion, raising concern for local recurrence. A core needle biopsy of the anterior chest wall lesion revealed SCNEC of unknown primary.

Slides from a previous mastectomy specimen, reported as poorly differentiated invasive ductal carcinoma (IDC), were reviewed with additional ancillary workup, revealing the same immunomorphology as the anterior chest wall lesion. Ipsilateral axillary node involvement (11/12) and the absence of a non-mammary NEC favored the breast as the primary site of the patient’s newly diagnosed SCNEC. She received a four-cycle regimen of carboplatin/etoposide/atezolizumab, the standard therapy for both advanced small-cell lung cancer and extrapulmonary SCNECs. While on maintenance therapy with atezolizumab, a positron emission tomography/computed tomography (PET/CT) in June 2020 revealed resolution of the anterior chest wall lesion but interval development of a left breast mass.

**Figure 2:**
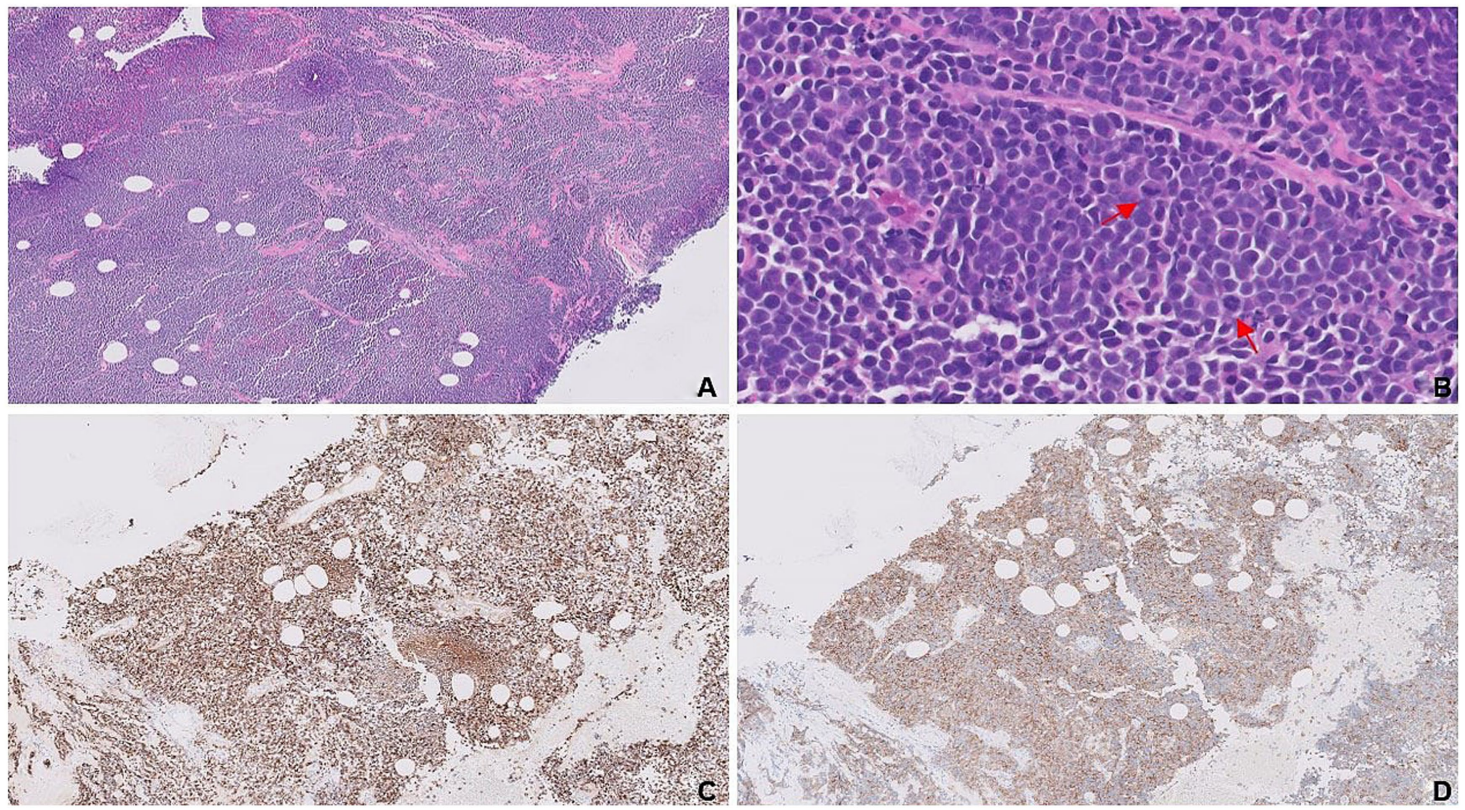
Complete effacement of the breast tissue by sheets of small blue tumor cells **(A)**. Dyscohesive, small, blue round cells with frequent mitotic figures (red arrows; **B**). Expressions of synaptophysin **(C)** and CD-56 **(D)** are confirmatory for neuroendocrine differentiation.

Microscopic examination of the left breast mass revealed a poorly differentiated neoplasm arranged in solid sheets effacing the breast parenchyma (Figure 2A). Tumor cells were characterized by their high nuclear-to-cytoplasmic (N: C) ratio, molded nuclei with “smudged” chromatin, absence of inconspicuous nucleoli, and frequent mitoses (Figure 2B). Immunohistochemical evaluation confirmed neuroendocrine differentiation by positive expressions of synaptophysin (Figure 2C), CD-56 (Figure 2D), and INSM1. Basal-like subtype was evidenced by HR-/HER2- immunophenotype, accompanied by cytoplasmic staining of tumor cells with the basal cell marker CK5/6. Ki-67 proliferation index was high, up to 40%. Molecular testing by targeted NGS revealed a total of six alterations. Four of these involved frequently implicated genes of SCNECs from various organs (e.g., lung, pancreas, and large bowel): *TP53, RB1, KMT2D,* and *KRAS* (9, 10). Additionally, two targetable alterations were detected in *AKT1* and *FGFR1* genes, both of which have been previously described in NEBCs (11, 12).

After exhausting the second-line therapy option with lurbinectedin, the patient was referred to a clinical trial of the pan-AKT inhibitor AZD5363. After 5 months of progression-free survival, the patient relapsed, prompting the initiation of hospice care in May 2021.

### 3.3 Case 3

A 73-year-old woman with a recent diagnosis of breast cancer presented with radicular back pain concerning for metastatic spinal cord compression. In addition to the dominant left breast mass, a recent PET/CT showed innumerable sclerotic/lytic lesions throughout the axial and proximal skeleton, consistent with bony metastases, as well as with mediastinal, hilar, and left axillary lymphadenopathy. The left breast lesion was initially reported as an HR+/HER2+ poorly differentiated IDC, while a recent fine needle aspiration biopsy of mediastinal lymph nodes came positive for SCNEC. A lumbar spine biopsy was performed to determine whether the bony lesions represented metastases from the breast or the lung, given that the majority of SCNECs originate in the lungs.

**Figure 3:**
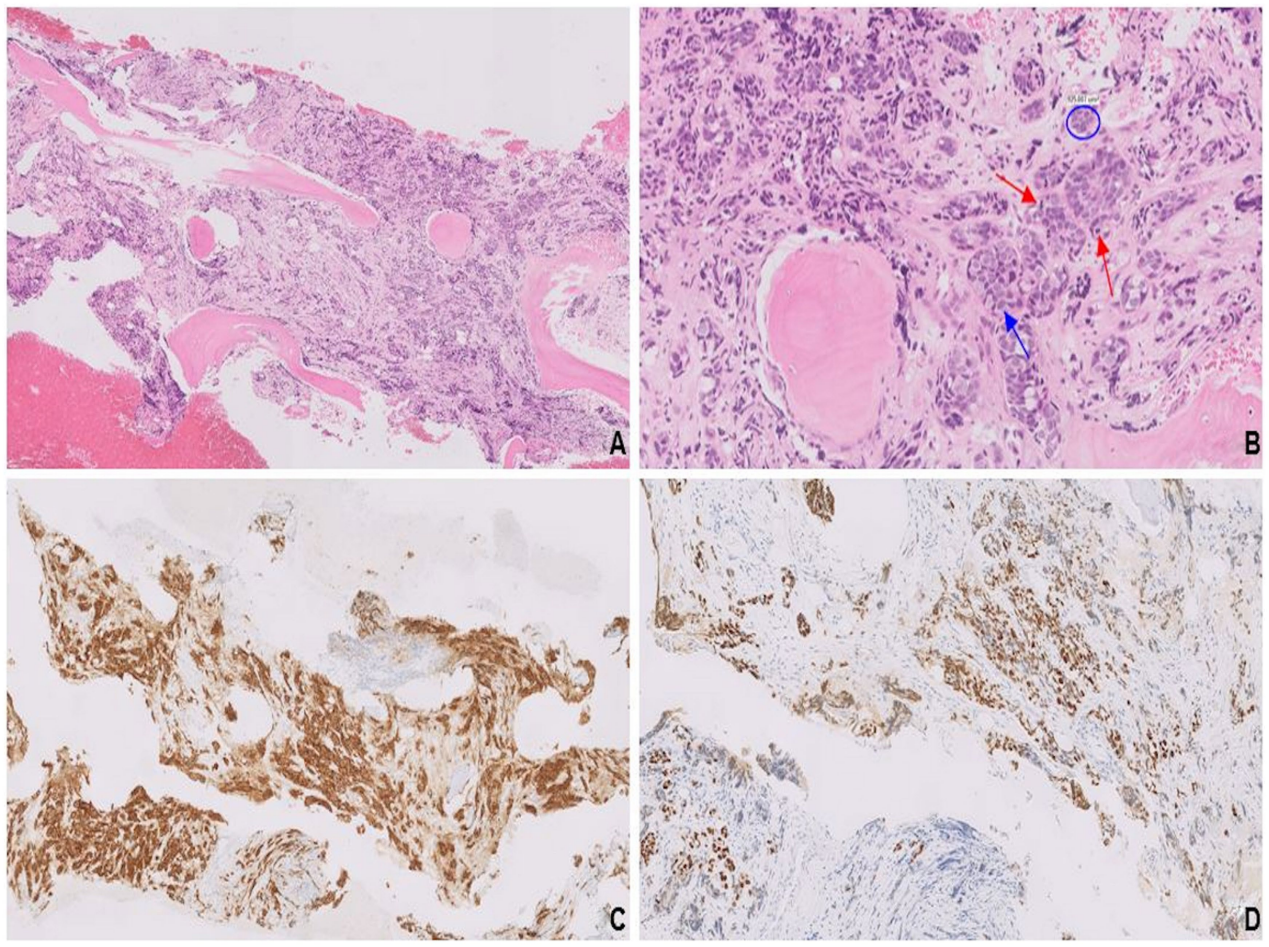
Solid nests and trabeculae of small round blue cells with crush artifact **(A)**. Tumor cells characterized by their scant cytoplasm, nuclear molding (blue circle), finely stippled chromatin (blue arrow), and frequent mitosis (red arrows; **B**). Synaptophysin expression **(C)** confirms neuroendocrine differentiation, and diffuse, strong immunoreactivity for GATA3 **(D)** is consistent with mammary origin.

The tumor was composed of solid nests and trabeculae of small round blue cells exhibiting “crush artifact” (Figure 3A). Cytologically, the tumor cells displayed molded nuclei with stippled chromatin (Figure 3B). A high proliferation rate was evidenced by frequent mitoses and a Ki-67 index up to 50%. Expression of neuroendocrine markers such as synaptophysin (Figure 3C), INSM1, and CD56 confirmed neuroendocrine differentiation, while negative p40 ruled out poorly differentiated squamous cell carcinoma. Diffuse, strong immunoreactivity for GATA3 (Figure 3D), along with negative staining for TTF-1, CDX-2, and PAX8, was consistent with mammary origin. Based on their HR+/HER2+ immunoprofile, the tumor cells belonged to the luminal B-like subtype, consistent with the patient’s breast cancer, which had initially been misclassified as poorly differentiated IDC. Diffuse immunoreactivity for synaptophysin confirmed neuroendocrine differentiation in the primary left breast lesion.

NGS analysis identified clinically significant amplifications of *ERBB2*, *FGFR2*, *CCNE1*, *MYC*, and *PPARG* genes, as well as a clinically significant variant in the *TP53* gene.

In January 2019, she began the first-line therapy for HER2 + metastatic breast cancer with a combination of pertuzumab, trastuzumab, and paclitaxel, which was later replaced by endocrine therapy with anastrozole due to paclitaxel-induced peripheral neuropathy. As of April 2024, the patient remained alive, albeit with radiographic progression of osseous metastases and the appearance of new pulmonary nodules.

### 3.4 Case 4

A 73-year-old woman was diagnosed with triple-negative, poorly differentiated IDC of the right breast at the 2 o’clock position. She underwent a lumpectomy, followed by a course of radiotherapy, completed in September 2012.

In April 2020, a focal right breast asymmetry was detected on annual surveillance mammography. A core needle biopsy showed a high-grade malignancy with areas of extensive necrosis. Diffuse immunoreactivity for synaptophysin was indicative of neuroendocrine differentiation, while negative staining for p63, S100, and SMA/desmin ruled out squamous, melanocytic, and myogenic differentiation, respectively. Positive expressions of GATA3, mammaglobin, and E-cadherin confirmed mammary origin, while negative staining for TTF-1, CDX2, and PAX8 excluded bronchopulmonary, gastrointestinal, and pancreatic primaries, respectively. Further ancillary work-up revealed a HER2-negative, luminal B-like phenotype, characterized by positive ER, low PR, and high Ki-67.

**Figure 4:**
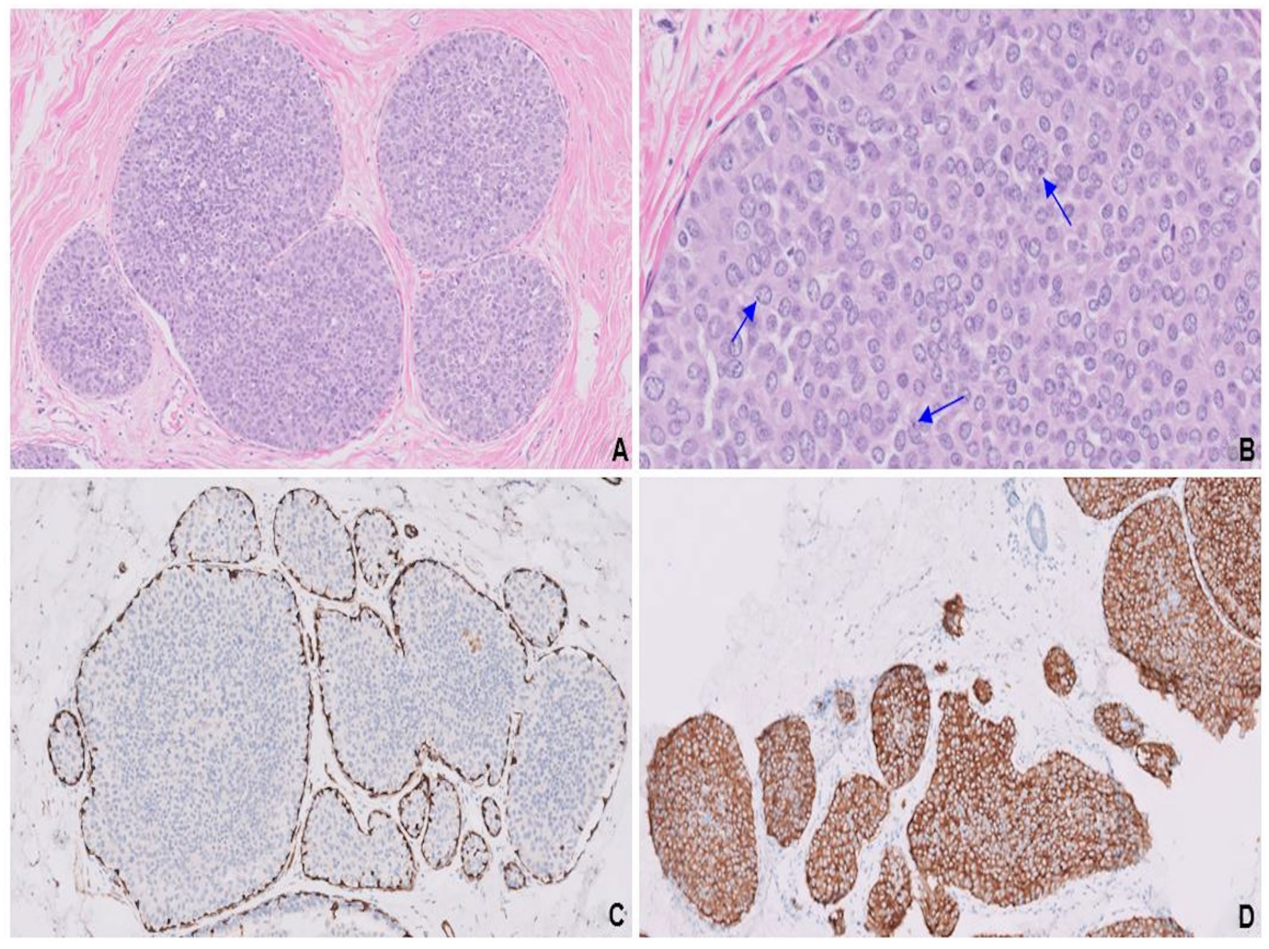
Endocrine ductal carcinoma *in situ* (E-DCIS) consisting of a duct expanded by the proliferation of monotonous cells **(A)**. Cells with abundant eosinophilic cytoplasm, vesicular nuclei, finely stippled chromatin, and conspicuous nucleoli (blue arrows; **B**). SMA highlights the intact myoepithelial cell layer **(C)** surrounding the intraductal component, while synaptophysin is uniformly expressed in both invasive and *in situ* components **(D)**.

A completion mastectomy specimen showed pT1cN0 LCNEC with an intraductal component (Figures 4A,B), providing compelling histological evidence for primary breast origin. SMA stain (Figure 4C) highlighted the intact myoepithelial cell layer surrounding the intraductal component. Both the invasive and intraductal components showed strong expression of synaptophysin (Figure 4D). Molecular analysis revealed no known clinically actionable alterations.

As of December 2023, antihormonal therapy was still being continued, and the patient is in good general health, free from tumor recurrence.

### 3.5 Case 5

**Figure 5:**
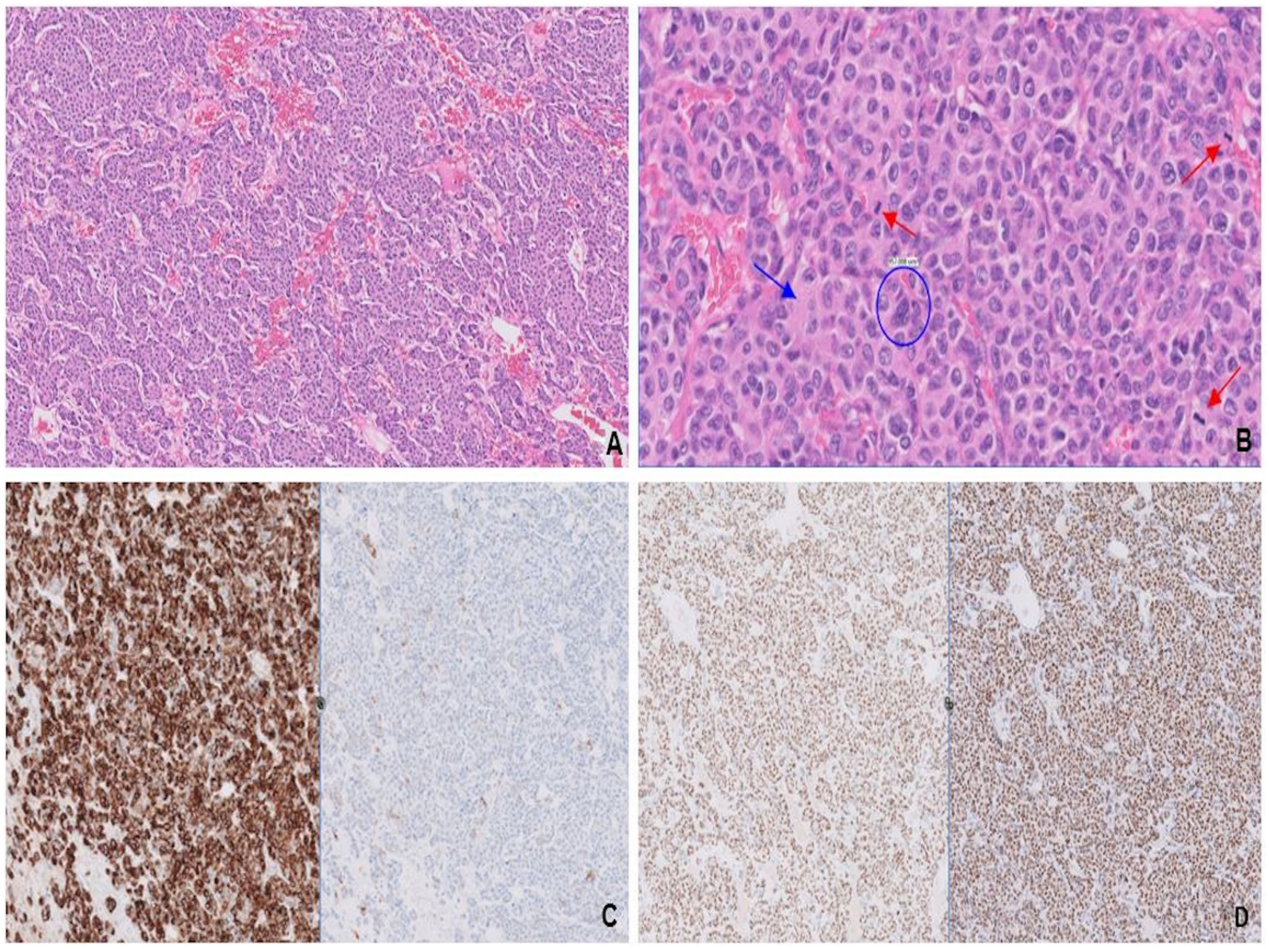
Variably sized solid nests separated by thin fibrovascular septae **(A)**. Tumor cells have abundant eosinophilic cytoplasm (blue arrow), pleomorphic nuclei with “salt-and-pepper” chromatin (blue circle), and variably conspicuous nucleoli, most consistent with large cell neuroendocrine carcinoma. Mitotic figures (red arrows; **B**) are frequent. Strong chromogranin (left) and patchy synaptophysin (right; **C**) expression confirms neuroendocrine differentiation, while expressions of ER (left) and GATA-3 (right; **D**) are consistent with breast origin.

A 27-year-old woman with a 2-year history of HR-positive/HER2-negative metastatic breast cancer presented with progressive, right frontal headaches over the past 2 months. Magnetic resonance imaging (MRI) of the brain revealed a large dural-based mass along the right frontal convexity, with an associated leftward shift across the midline. The presence of additional parenchymal lesions raised concern for metastases rather than a primary brain tumor. The right frontal mass was excised to relieve the increased intracranial pressure. Pathology revealed variably sized solid nests and rows, separated by thin fibrovascular septae (Figure 5A). Tumor cells displayed abundant eosinophilic cytoplasm and pleomorphic nuclei with stippled chromatin (Figure 5B), suggestive of LCNEC. Expression of all four neuroendocrine markers [patchy synaptophysin, diffuse and strong chromogranin expression (Figure 5C), and diffuse and strong staining for INSM1 and CD56 (not shown)] was compelling for neuroendocrine differentiation.

Tumor cells exhibited a CK7+/CK20- profile with a wide differential for the site of origin, including breast, mullerian, lung, thyroid, upper gastrointestinal, and pancreatobiliary tract carcinomas. Considering the past medical history of breast cancer and negative clinical workup for another primary, the breast was considered the most probable primary tumor site. This was supported by uniform expressions of ER and GATA-3 (Figure 5D), as well as high PR expression (20%), favoring a luminal A-like phenotype. The NGS analysis showed three alterations involving the frequently implicated genes of HR-positive breast cancer: *PIK3CA, TP53,* and *MAP2K4* (8). Additionally, a recurring alteration was identified in *FGFR1* gene, the amplification of which was recently shown in NEBCs (12).

Given the progression on prior endocrine therapy, palbociclib was added to anti-hormonal therapy with fulvestrant and goserelin. As of May 2020, the patient was still alive with radiographic progression of intracranial metastases, as well as the appearance of new liver lesions.

### 3.6 Case 6

A 49-year-old woman with no significant history presented after a syncopal fall. Associated signs/symptoms included 3 weeks of progressive weakness, confusion, and lethargy, as well as an unintentional weight loss of 40 pounds over the past 6 months. She denied any personal/family history of malignancy; however, she reported having an abnormal screening mammography of her left breast 4 years ago, which she did not follow up with. MRI of the brain demonstrated a 3.3 cm left frontal cavitary mass with rim enhancement. Numerous subcentimeter metastatic deposits were noted within the bilateral cerebral/cerebellar hemispheres.

**Figure 6:**
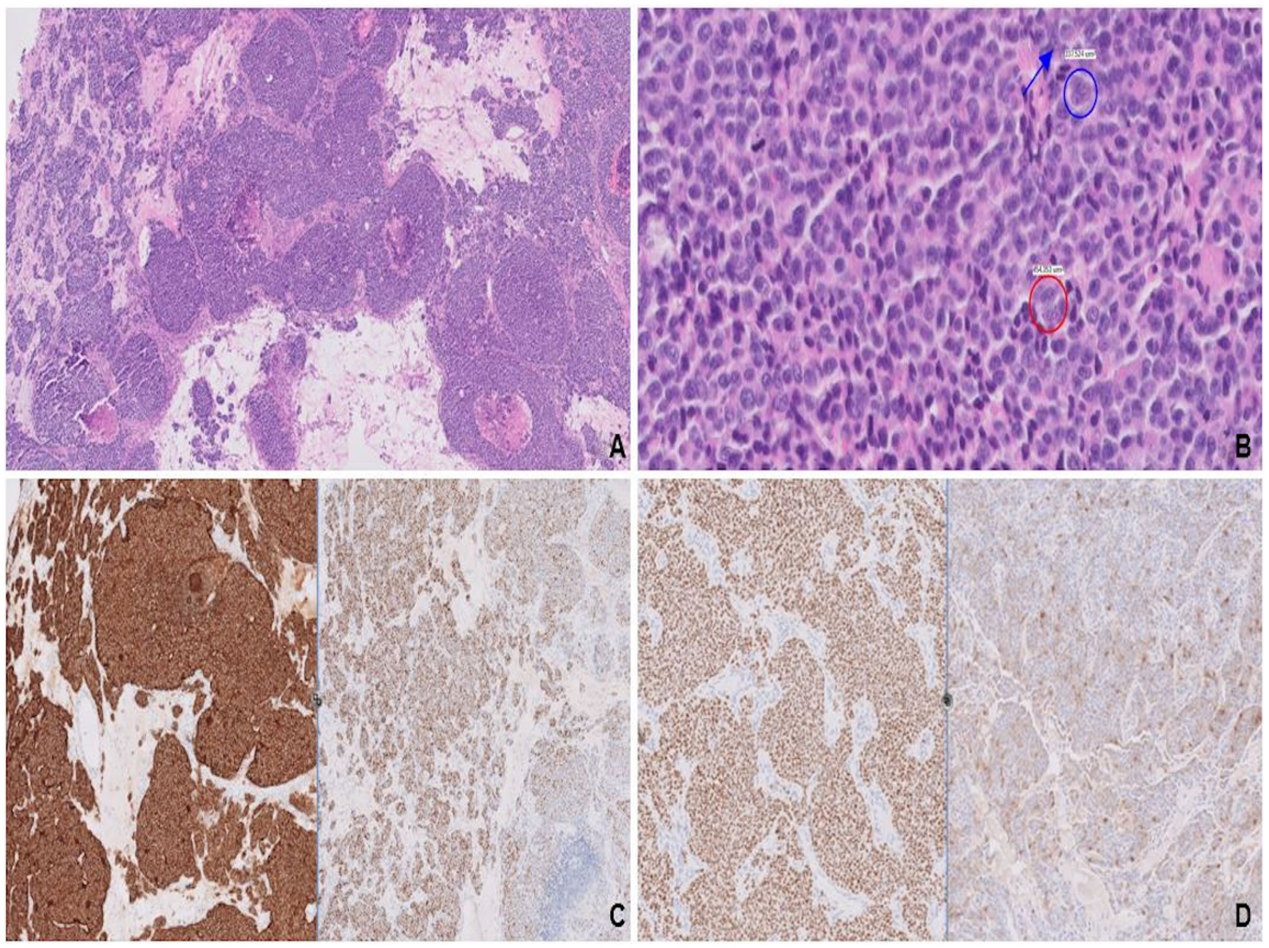
Variably sized solid nests with areas of central comedonecrosis **(A)**. Nuclear features, stippled chromatin (red circle) with conspicuous nucleoli (blue circle and arrow; **B**), most consistent with large cell neuroendocrine carcinoma. Strong and diffuse expression of synaptophysin (left) along with patchy, strong staining for INSM1 (right; **C**), confirms neuroendocrine differentiation. Mammary origin is evidenced by diffuse, strong staining for GATA3 (left) and patchy staining for BRST-2 (right; **D**).

A whole-body CT scan showed a heterogeneously enhancing left breast mass extending to the anterior chest wall and skin surface. In addition to the solitary left breast lesion, extensive metastases were identified, involving the pleura/pericardium, bilateral lungs, liver, and spine. Left frontal craniotomy revealed a high-grade malignancy with nested architecture and areas of central comedo-necrosis. (Figure 6A). Salt-and-pepper chromatin with conspicuous nucleoli (Figure 6B) and abundant eosinophilic cytoplasm was suggestive of LCNEC. Diffuse expression of synaptophysin (Figure 6C, left), chromogranin, and CD56, along with patchy but strong staining for INSM1 (Figure 6C, right), confirmed neuroendocrine differentiation. Uniform expressions of ER and GATA3 (Figure 6D, left) as well as patchy staining for BRST2 (Figure 6D, right) were indicative of mammary origin. The lack of staining for TTF-1, CDX2, and PAX8 excluded bronchopulmonary, gastrointestinal, and pancreatic primaries, respectively. Low PR expression (5%) with a high Ki-67 (40%) favored a luminal-B-like phenotype. Staining for membranous HER2 was negative.

Molecular testing revealed a clinically significant variant in *TP53* gene and amplification of the *FGF3* and *CCND1* genes, the latter of which was previously reported in NEBC (12).

Following left frontal craniotomy, antihormonal therapy with tamoxifen was initiated. However, the patient declined palliative chemotherapy given her baseline poor performance status. Hospitalization was complicated by obstructive pneumonia with severe sepsis, leading to her death a month after her initial diagnosis of widely metastatic NEBC.

## 4 Discussion

### 4.1 Current terminology, classification, and epidemiology of primary neuroendocrine neoplasms of the breast

Neuroendocrine differentiation in breast carcinomas was first described in mucinous carcinomas by Feyrter and Hartmann in 1963 (13). The first case series was published in 1977, introducing the term “primary carcinoid of the breast” (14). In third edition of the WHO classification (2003), NEBCs were first recognized as a distinct entity, defined as epithelial tumors morphologically resembling NENs of the gastrointestinal tract and lung, with >50% of the tumor expressing neuroendocrine markers (3).

The 2012 WHO Working Group categorized mammary NENs into two major groups: well-differentiated NETs and poorly-differentiated small cell neuroendocrine carcinomas (SCNECs) (1). By this definition, NEBCs included SCNECs but not LCNECs. A third category encompassed conventional breast carcinomas with neuroendocrine differentiation, as well as special subtypes of breast carcinoma, such as solid papillary carcinoma and the hypercellular variant of mucinous carcinoma. The 2019 WHO classification revised this framework by excluding the latter subtypes and formally including LCNECs (6).

NEBCs are rare, with their true incidence likely underestimated due to frequent misclassification as poorly-differentiated IBC-NST/IDC, other histologic subtypes, or metastatic NEC (15). Their incidence among all types of breast carcinoma ranges from 0.1 to 5%. Based on the 2012 WHO criteria, mammary NENs account for 2–5% of cases (1). However, this representation likely included WDNETs. An analysis of surveillance, epidemiology and end results (SEER) registry data from 2003 to 2009 by Wang et al. identified only 142 NEBC cases, approximating to 0.1% of all breast cancers (2).

### 4.2 Clinical aspects

NEBCs lack distinctive clinical features, making diagnosis challenging. Compared to IDC, patients more often present with stage II disease with an increased propensity for regional lymph node involvement (16).

NEBCs typically affect white, postmenopausal women in their 60s and 70s, although rare cases occur in premenopausal women (17) and men (18, 19). Compared to IBC-NST, NEBCs present at an older age with larger tumors, higher histologic grade, and more advanced stage. In our series, the mean tumor size was 48 mm (range 17–95 mm), which was more than double the 23 mm mean tumor size reported for IBC-NSTs (2).

Imaging features are non-specific. Gallo et al. described the most common mammographic finding as a hyperdense, irregular, solitary mass (20). To rule out an extra-mammary primary, CT of the chest and abdomen is recommended. Gallium-68 PET/CT may aid in well-differentiated cases, while FDG PET/CT is preferred for poorly differentiated NEBCs.

### 4.3 Histological diagnosis and ancillary studies

In the latest WHO classification, NEBCs are defined by high-grade morphology resembling their pulmonary counterparts. SCNECs show infiltrative solid sheets of densely packed, hyperchromatic cells with high N:C ratios, scant cytoplasm, and inconspicuous nucleoli. In contrast, LCNECs exhibit abundant cytoplasm and pleomorphic nuclei with prominent nucleoli.

NEBCs are often underrecognized due to the absence of classic neuroendocrine features such as “salt-and-pepper” chromatin (21). They may mimic poorly differentiated IDC, invasive lobular carcinoma, or solid DCIS. Accurate diagnosis requires careful morphologic evaluation followed by IHC confirmation. Among neuroendocrine markers, synaptophysin shows the highest sensitivity. Additional markers—INSM1, chromogranin, and CD56—have variable expression. Neuron-specific enolase is non-specific and currently, it is not recommended as part of the diagnostic panel. Recently, a tissue microarray analysis showed that adding chromogranin to synaptophysin detects an extra 4.2% of breast cancer cases with neuroendocrine differentiation, while INSM1 identifies 15% of cases negative for both (22). INSM1 expression has also been associated with improved disease-free survival in luminal breast cancers, supporting its inclusion alongside synaptophysin ± chromogranin in diagnostic panels (23).

Given their rarity, metastatic NECs must first be excluded. The presence of an *in situ* component confirms breast origin, although it is rarely observed in biopsies. Lineage-specific markers aid in distinction: ER, GATA3, mammaglobin, and GCDFP15 support a mammary origin, while TTF-1, CDX2, and PAX8/Islet 1 help exclude pulmonary, gastrointestinal, and pancreatic primaries, respectively.

Unlike gastrointestinal or pulmonary NENs, mammary NENs are not formally graded by the Ki-67 index, but Ki-67 remains useful for risk stratification, similar to its role in luminal-type breast cancer (24).

### 4.4 Current management and prognosis

There are no established treatment guidelines for NEBCs and their management largely mirrors that of ductal-type breast cancer. Early-stage disease is treated with surgery +/− radiotherapy based on tumor size and nodal status. Chemotherapy is used for metastatic disease or as neoadjuvant therapy in locally advanced, inoperable cases (25). Combinations of platinum agents and etoposide—as used for pulmonary/extra-pulmonary SCNECs—are commonly administered alongside taxane-based chemotherapy, which is routinely used for breast cancer (26). In our cohort, the patient with triple-negative SCNEC was initially treated with taxane-based chemotherapy, followed by a platinum-etoposide combination.

The majority of NEBCs are HR-positive, supporting the use of endocrine therapy. In cases of endocrine therapy resistance, the addition of CDK4/6 inhibitors (e.g., palbociclib) can improve outcomes in HR-positive disease by targeting cell-cycle pathways (27). All HR-positive patients in our series received endocrine therapy. In one case of endocrine resistance, CDK 4/6 inhibitor palbociclib was administered along with conventional endocrine therapy (i.e., letrozole). Anti-HER2 therapy may be considered in rare HER2-amplified NEBCs, as in our case with HER2 overexpression (28).

Emerging data suggest that somatostatin receptors (SSTR2A and SSTR5) may be expressed in up to 71% of tumors, indicating a potential therapeutic target analogous to other NENs (29).

Prognosis remains controversial, likely due to evolving classification. Earlier WHO definitions included indolent subtypes (e.g., solid papillary and hypercellular mucinous carcinomas), which may have obscured the outcome data. However, recent studies suggest poorer outcomes. An analysis of 142 NEBCs from the SEER database demonstrated worse overall and disease-free survival compared to IBC-NST (2). Multivariate analysis confirmed neuroendocrine differentiation as an independent adverse prognostic factor. NEBCs also show a high risk of recurrence, with distant metastases reported in the liver, bone, lungs, pleura, brain, pancreas, and soft tissues (16, 25, 30). In our series, 83% (5/6) of patients developed distant metastases within 4 years of diagnosis.

Given their aggressive behavior and potential for delayed metastasis years after treatment, long-term radiologic surveillance is recommended.

## 5 Conclusion

NEBCs are rare tumors that typically exhibit an ER-positive, luminal phenotype. However, unlike IBC-NST, where the luminal phenotype correlates with favorable prognosis, NEBCs are associated with poorer outcomes at similar stages (2, 30).

In this study, molecular profiling of six NEBC cases revealed potentially actionable alterations in three cases: *FGFR1* amplifications (cases 2 and 5) and PI3K/AKT/mTOR pathway alterations (cases 1, 2, and 5). Although comprehensive genomic data on NEBCs remain limited, recurrent aberrations in FGF/FGFR and PI3K/AKT/mTOR pathways have been reported (31). The presence of PI3K/AKT/mTOR alterations suggests molecular overlap with IBC-NST (32).

These pathways offer therapeutic opportunities. Everolimus, an mTOR inhibitor, is FDA-approved for use in both pancreatic NENs and HR-positive breast cancer in combination with exemestane (33, 34). Other agents include PI3K inhibitors and the pan-AKT inhibitor AZD5363, which was used in a triple-negative small cell NEBC case in our cohort (Case #2), achieving 5 months of progression-free survival.

The FGF/FGFR pathway also represents a potential target for precision therapy, with selective FGFR inhibitors showing efficacy in tumors with FGFR mutations, amplifications, or gene fusions (35, 36), including single-agent activity in breast carcinoma (37).

Consideration of NEBCs in a differential diagnosis of poorly differentiated breast cancer is essential for timely and accurate diagnosis, which, in turn, can facilitate optimal management, including the application of aforementioned precision oncology approaches.

## Data Availability

The original contributions presented in the study are included in the article/supplementary material, further inquiries can be directed to the corresponding author.

## Glossary

NEBC: neuroendocrine breast carcinomas
NEC: neuroendocrine carcinoma
SCNEC: small cell neuroendocrine carcinomas
LCNEC: large cell neuroendocrine carcinomas
IBC-NST: invasive breast carcinoma of no special type
IDC: invasive ductal carcinoma
HR: hormone receptor
NENs: neuroendocrine neoplasms
IHC: immunohistochemistry
NET: neuroendocrine tumors
CT: computed tomography
PET/CT: positron emission tomography/computed tomography
MRI: magnetic resonance imaging
ER: estrogen receptor
PR: progesterone receptor
HER2: human epidermal growth factor receptor 2
N: C: nuclear-to-cytoplasmic
NGS: next-generation sequencing
SEER: surveillance, epidemiology and end results
PI3K/AKT/mTOR: phosphoinositide 3 kinase/AKT/mammalian target of rapamycin.
